# Mulberry leaf polysaccharide improves cyclophosphamide-induced growth inhibition and intestinal damage in chicks by modulating intestinal flora, enhancing immune regulation and antioxidant capacity

**DOI:** 10.3389/fmicb.2024.1382639

**Published:** 2024-03-21

**Authors:** Ming Cheng, Yongbin Shi, Yumeng Cheng, Hongjie Hu, Song Liu, Yanping Xu, Lingzhi He, Shanshan Hu, Yujie Lu, Fengmin Chen, Jiang Li, Hongbin Si

**Affiliations:** ^1^College of Animal Science and Technology, Guangxi Key Laboratory of Animal Breeding, Disease Control and Prevention, Guangxi University, Nanning, China; ^2^Hunan Provincial Key Laboratory of the TCM Agricultural Biogenomics, Changsha Medical University, Changsha, China

**Keywords:** mulberry leaf polysaccharide, oxidative stress, immunosuppression, intestinal damage, growth performance, intestinal flora

## Abstract

Polysaccharides are generally considered to have immune enhancing functions, and mulberry leaf polysaccharide is the main active substance in mulberry leaves, while there are few studies on whether mulberry leaf polysaccharide (MLP) has an effect on immunosuppression and intestinal damage caused by cyclophosphamide (CTX), we investigated whether MLP has an ameliorative effect on intestinal damage caused by CTX. A total of 210 1-day-old Mahuang cocks were selected for this experiment. Were equally divided into six groups and used to evaluate the immune effect of MLP. Our results showed that MLP significantly enhanced the growth performance of chicks and significantly elevated the secretion of cytokines (IL-1β, IL-10, IL-6, TNF-α, and IFN-γ), immunoglobulins and antioxidant enzymes in the serum of immunosuppressed chicks. It attenuated jejunal damage and elevated the expression of jejunal tight junction proteins Claudin1, Zo-1 and MUC2, which protected intestinal health. MLP activated TLR4-MyD88-NF-κB pathway and enhanced the expression of TLR4, MyD88 and NF-κB, which served to protect the intestine. 16S rDNA gene high-throughput sequencing showed that MLP increased species richness, restored CTX-induced gut microbiome imbalance, and enhanced the abundance of probiotic bacteria in the gut. MLP improves cyclophosphamide-induced growth inhibition and intestinal damage in chicks by modulating intestinal flora and enhancing immune regulation and antioxidant capacity. In conclusion, this study provides a scientific basis for MLP as an immune enhancer to regulate chick intestinal flora and protect chick intestinal mucosal damage.

## 1 Introduction

Poultry are raised intensively, and chicks are often affected by stressful conditions, viral infections, nutritional deficiencies, infectious diseases, and other conditions that can cause immunosuppressive diseases (Shini et al., 2010). It not only affects the immune function of chicks, but also causes symptoms such as intestinal damage and intestinal oxidative stress, which also reduces the conversion rate of chick feed and affect the feed-to-meat ratio (Fussell, 1998; Jahanian and Rasouli, 2015). In the state of immunosuppression, the body loses the ability to resist pathogens, leading to a sharp increase in morbidity and mortality among chicks, which causes substantial losses in the farming industry (Hoerr, 2010).

Cyclophosphamide (CTX), the broad-spectrum chemotherapeutic utilized to treat cancer, kills cancer cells primarily through the genotoxicity and cytotoxicity of drug (Emadi et al., 2009). However, the excessive use of CTX can also cause body immunosuppression and oxidative stress, liver damage, and intestinal injury caused by intestinal mucosal barrier disruption (Duncan and Grant, 2003; Ahlmann and Hempel, 2016; Zheng et al., 2017). The intestine is the largest digestive organ of the body and also the greatest immune organ, while the intestinal barrier and mucosal immune system represent a key system to maintain body health against external pathogens (Walker et al., 2014). Therefore, in the case of declined immune function, the intestinal barrier and intestinal mucosal immune system are destroyed, the disease resistance of the body will be decreased and the risk of disease will be increased (Schoultz and Keita, 2019). In addition, under normal circumstances, the intestinal tract of the body is rich in flora to maintain the dynamic balance of the intestine, but is highly susceptible to the influence of the external environment, causing diarrhea and intestinal inflammation (Jang et al., 2019). Therefore, it is necessary to develop natural pharmaceutical feed additives that can increase organism immunity and improve intestinal flora and function (Wu et al., 2017; Wang et al., 2019).

Polysaccharides, as a type of high molecular weight long-chain carbohydrates, are widely present in plants, animals, and microorganisms (Kong et al., 2004). It has functions such as enhancing immunity and antioxidant and anti-inflammatory properties (Yu et al., 2018). Meanwhile, a large number of experiments have also proven that polysaccharides have the effect of improving the growth performance of livestock and enhancing immunity (Shu et al., 2021; Zhang et al., 2021). Mulberry leaf polysaccharide (MLP) is a natural polymer extracted from mulberry leaves and has been proven to have good antioxidant activity (Yuan et al., 2015). Mulberry leaf polysaccharide can also antagonize diabetes (Zhang et al., 2014). Meanwhile, Zhao demonstrated that MLP can improve the metabolism and immune function of weaned piglets (Zhao et al., 2019). However, the role of MLP in regulating intestinal injury and immune performance in immunosuppressive chicks is not clear. Therefore, this experiment established a CTX-induced immunosuppressive model in chicks to study the effects of MLP on the growth performance, immune performance, antioxidant performance, and intestinal injury of CTX-induced immunosuppressive chicks.

## 2 Materials and methods

### 2.1 Materials

We obtained mulberry leaf samples in the mulberry leaf garden in Nanning, Guangxi, China.

### 2.2 Preparation of MLP

After collection, fresh mulberry leaf samples were subjected to shade drying, crushing, and passing through 60 mesh sieve, after which they were soaked in 85% of ethanol for more than 7 days and then dried at 50°C. According to the 34 mL/g material–liquid ratio, at a 92°C extraction temperature, a 3.5-h extraction time, and after 2 extractions, the filtrate was combined by filtration and centrifugation, concentrated under reduced pressure at 70°C, the concentrate was centrifuged to remove the residue, anhydrous ethanol at 4-fold volume was introduced for concentration and left for 13 h, then the precipitate was collected by centrifugation, the precipitate was washed using anhydrous ethanol at 3-fold volume and collected by centrifugation again, and finally freeze-dried to obtain mulberry leaf polysaccharide. The content of sugar was 51.02% by phenol–sulfuric acid method.

### 2.3 Determination of physicochemical properties of the polysaccharide

Using the Shimadzu LC-10A system containing the BRT105-104-102 column (8 × 300 mm, Borui Saccharide) and the parallax detector, Mw of MLP was analyzed with high-performance gel permeation chromatography (HPGPC). Calibration curves were also drawn to determine molecular weight. Fourier transform infrared spectroscopy (FT-IR) was then conducted to analyze organic functional groups within MLP. Later, dried polysaccharides were blended with KBr powder, followed by pressing the mixture to sheets to record using the Fourier transform infrared spectrometer FT-IR650 within 4,000–400 cm^−1^.

### 2.4 Preparation of MLE

Weigh a certain amount of dried mulberry leaves, add 20 times the weight of distilled water, soak for 2 hours, heat to boiling, and then keep boiling for 2 hours. Then filter the extract to remove the filter residue, centrifuge to remove impurities, use a rotary evaporator to concentrate the extract into 1 mL of liquid, equivalent to 1 g of the original drug, and then freeze dry it into powder using a freeze-drying machine. After drying, seal and store it.

### 2.5 Animal experiment

The Experimental Animal Ethics Committee of Guangxi University (GXU-2023-0013) approved our animal experimental protocols. In total, 210 1-day-old Mahuang cocks were chosen for the experiments. They were kept within wired cages with lighting and good ventilation at 50–55% relative humidity. For 1–14-day-olds, a 24-h light period was provided for the chicken house, followed by a gradual decline into 20 h every day. At 1–7-day-old, chicken house temperature was maintained under 32–34°C, followed by a gradual decline to 26°C. In this experimental process, chickens could eat and drink freely. After the chicks finished acclimatization, the chicks were evenly classified into 6 groups based on body weight at 7 days of age, with 7 chicks for every replicate, and 5 replicates for every group, specifically into blank control group (NC), cyclophosphamide model group (MC), mulberry leaf polysaccharide immunosuppression group (MLP + CTX), mulberry leaf aqueous extract immunosuppression group (MLE + CTX), normal mulberry leaf polysaccharide group (MLP), and normal mulberry leaf aqueous extract group (MLE), and the experimental days were 35 days. During this period, NC and MC groups were given the basal diet, the MLP + CTX and MLP groups added 0.25% MLP to the basal diet, and the MLE + CTX and MLE groups added 0.25% MLE to the basal diet. On days 29, 31, and 33, the MC, MLP + CTX, and MLE + CTX groups were given injections of 80 mg/kg of CTX into the thoracic muscle, and the NC, MLP, and MLE groups were given injections of normal saline at an equivalent volume. On days 14, 21, 28, and 35, feed intake and body weight were determined to calculate the meat and feed ratio. At 35 days, we obtained blood, thymus, spleen, bursa, jejunum, and cecum contents from the chicks for subsequent analysis (*n* = 6). Table 1 displays diet information.

**Table 1:** Basic diet information (%).

Items	Days 1–14	Days 15–28	Days 29–42
Ingredients			
Corn	56.80	57.36	58.88
Soybean meal	36.48	34.57	33.01
Soybean oil	1.63	3.42	3.89
NaCl	0.53	0.53	0.53
Limestone	1.40	1.22	1.05
CaHPO4	2.10	1.99	1.85
L-Lys·HCl	0.30	0.15	0.06
DL-Met	0.26	0.26	0.23
Choline chloride	0.10	0.10	0.10
Premix^1^	0.40	0.40	0.40
Total	100.00	100.00	100.00
Nutrient levels^2^			
AME, MJ/kg	12.89	13.50	13.94
Crude protein	21.00	20.00	20.00
Calcium	1.01	1.00	0.95
Available phosphorus	0.62	0.60	0.57
Lysine	1.04	0.91	0.88
Methionine	0.41	0.40	0.38
Threonine	0.74	0.70	0.70
Tryptophan	0.22	0.19	0.18

^1^The premix is provided per kilogram of ration: VA 10000 IU; VD 5200 IU, VE 40 IU; VK 8 IU; VB1 5 IU; VB2 8 IU; VB6 12 IU; VB12 2 IU; biotin 0.2 mg; nicotinic acid 45 mg; folic acid 0.8 mg; Fe 100 mg, Cu 8 mg, Zn 100 mg, Mn 120 mg, I 0.3 mg, Se 0.7 mg. ^2^Nutrient levels were calculated values. AME, apparent metabolizable energy.

### 2.6 Growth performance and index of spleen, thymus, and bursa of Fabricius determination

Through this experiment, we measured body weight and feed consumption of chicken every day, acquired their spleen, thymus, and BF, and recorded the weights at the end of this experiment. The index of the spleen, thymus, and BF was determined as follows:

Index (g/kg) = immune organ weight/body weight.

### 2.7 Measurements of cytokines and immunoglobulins

Blood samples were obtained into the sterile tube, followed by 10-min natural coagulation under ambient temperature (Yin et al., 2020) and an additional 20-min centrifugation (3,000 rpm under 4°C) to obtain the serum. At last, the ELISA kits (Nanjing Boyan Biotech Co) were utilized for detecting IL-1β, IL-6, IL-10, IFN-γ, TNF-α, and IgG levels.

### 2.8 Determination of serum antioxidant enzyme activity

The serum collection method is consistent with 2.7. Serum samples were obtained from superoxide dismutase (SOD), catalase (CAT), glutathione peroxidase (GPx), total antioxidant capacity (T-AOC), and malondialdehyde (MDA) levels using an ELISA kit (Nanjing Boyan Biotech Co).

### 2.9 Histopathological staining

Chick jejunal and ileal samples were subjected to overnight fixation using 10% neutral formaldehyde, followed by dehydration, pruning, embedding, sectioning, staining, and tablet sealing for microscopic examination (Deng et al., 2021). Later, the digital trim camera microscope (BA210 Digital, Motic) was utilized for section observation; meanwhile, the image analysis software (Motic Images Advanced 3.2) was employed for measuring and analyzing villus length, crypt depth, and intestinal wall of jejunal and ileal samples. The tissue of 4 chicks in each group was selected as slices, three fields of view were selected from each tissue section, and 10 villus heights and 10 crypt depths were measured in each field with Image-Pro Plus 6.0. The villus length-to-crypt depth ratio was determined to be an index for evaluating the degree of intestinal injury.

### 2.10 RNA isolation and RT-PCR assay

Frozen jejunal samples (50–100 mg) were ground within liquid nitrogen; later, the TRIGene total RNA extraction reagent was utilized for RNA extraction. cDNA was synthesized using StarScript II First-Strand cDNA Synthesis Mix (GenStar) with gDNA remover. By adopting RealStar Green Fast Mixture, PCR was carried out using the LightCycler 96 System (Roche). RT-PCR was then conducted to detect TLR4, MyD88, NF-κB, Occludin, MUC2, ZO-1, IL-10, and IFN-γ gene levels, with β-actin being the endogenous control. The 2-11CT approach was utilized to determine relative gene expression. Supplementary Table S1 displays primer sequences utilized in this study.

### 2.11 DNA isolation from intestinal flora and 16S rDNA gene high-throughput sequencing

The DNA Kit (Omega) was utilized for DNA extraction from cecal contents. Universal primers 341F (5’-CCTAYGGGRBGCASCAG-3′) and 806R (5’-GGACTACNNGGGTATCTAAT-3′) were utilized to amplify V3-V4 hypervariable regions of the bacterial 16S rDNA gene. The AxyPrep DNA Gel Recovery Kit (Axygen) was utilized to purify PCR products, whereas QuantiFluor™-ST (Promega) was used for quantification. The TruSeCT60M DNA Sample Prep Kit was employed for constructing the PE library, while the Illumina MiSeq PE300 platform (Illumina) was applied in sequencing in line with specific protocols. UPARSE software was later used in high-quality sequence clustering for obtaining operational taxonomic units (OTUs) according to the 97% similarity level. Alpha- and beta-diversities were examined in line with OTU abundances with the R package. For predominant bacterial species at the phylum and genus levels, their relative abundances were determined. Between-group dominance of microbial communities was analyzed through linear discriminant analysis (LDA) and linear discriminant analysis effect size (LEFSE). Several groups were compared by one-way ANOVA, while two groups were compared by Wilcoxon rank-sum tests.

Note: In the sequencing results of 16S rDNA in this section, in all tables and figures, MLP represents MLP + CTX, while MLE represents MLE + CTX, which means injection of CTX is used to evaluate the improvement effect of MLP and MLE on CTX-induced intestinal dysbiosis. NC group still represents the blank control group, and MC group represents the cyclophosphamide injection group.

### 2.12 Statistical analysis

Experimental data within tables and figures were represented by mean ± SD, while between-group differences were evaluated through one-way ANOVA and Tukey’s multiple comparisons with SPSS. GraphPad Prism 6.0 was applied in graph plotting. Majorbio cloud platform was employed for analyzing 16S rDNA sequencing data. *p* < 0.05 and *p* < 0.01 represent significant and extremely significant differences, separately.

## 3 Results

### 3.1 Physicochemical properties of MLP

**Figure 1:**
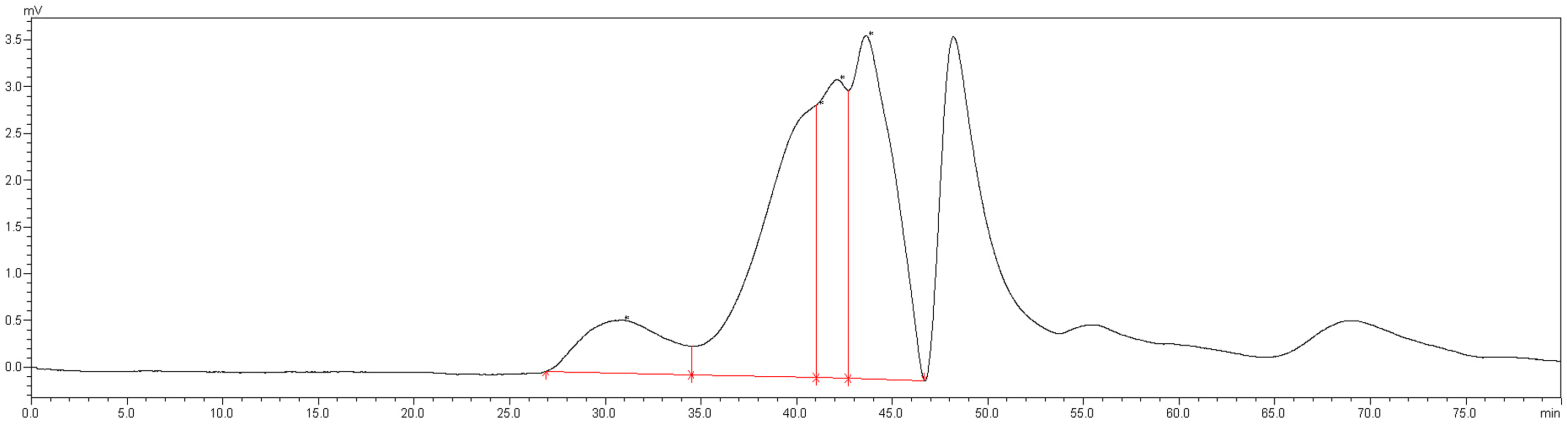
Molecular weight detection results of MLP.

**Figure 2:**
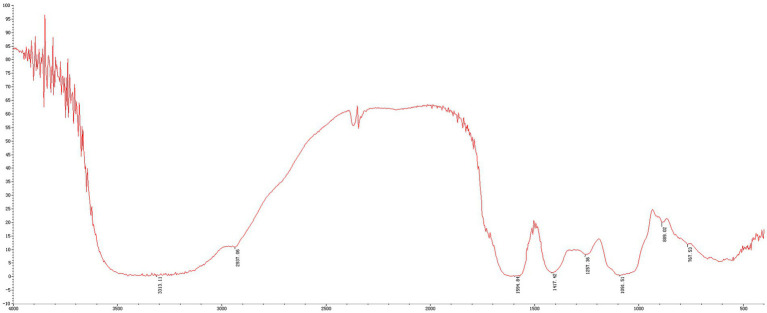
FT-IR spectra of MLP.

The MLPs utilized in this study have fundamental features and physicochemical properties of polysaccharides. The Mw values were determined to be 758.773 kDa, 11.968 kDa, 7.643 kDa, and 4.149 kDa, respectively (Figure 1). According to the above findings, there were various polysaccharides within MLP (Figure 2). The absorption band at 3600–3200 cm-1 was ascribed to -OH stretching vibration; typically, absorption peaks within the above region represented the typical peaks of sugars. Later, the peak at 3313 cm-1 stands for an absorption peak related to O-H stretching vibration, and it was the typical peak of sugars. One absorption peak could be detected at 2937 cm-1, probably associated with C-H stretching vibration. The absorption peak detected at 889 cm-1 was associated with C-H variable angle vibration of β-terminus differential isomerization of the pyran ring. The absorption peak detected at 767 cm-1 was associated with the symmetric ring stretching vibration of the pyran ring. Based on the above findings, MLP shows typical adsorption of characteristic polysaccharides.

### 3.2 Effect of MLP on growth performance and the immune organ index in chicks

According to our average daily gain (ADG), average daily feed intake (ADFI), and feed conversion ratio (FCR) results, MLE did not significantly differ in ADG, ADFI, and FCR at all three stages (*p* > 0.05). However, MLP apparently elevated ADG (*p* < 0.05) but significantly declined FCR (*p* < 0.05) of chickens during D7-D21, and MLP highly significantly decreased FCR in chickens during D21-D28 (*p* < 0.01), while during D28-D35, ADG was highly significantly increased (*p* < 0.001), but FCR was extremely markedly reduced (*p* < 0.001) of MLP-treated group relative to NC. In addition, we examined the changes in chick growth performance before and after CTX injection during D28-D35. According to these findings, CTX administration markedly decreased ADG and elevated FCR (*p* < 0.001) in chicks, and the application of MLP significantly restored ADG in chicks (*p* < 0.05) but evidently decreased FCR of the MC group (*p* < 0.05; Tables 2, 3).

**Table 2:** Effect of MLP on the growth performance of chicks.

Items	NC	MLP	MLE	*p*-value
D7-D21				
ADG, g/d	3.5 ± 0.19	4.05 ± 0.13^*^	3.77 ± 0.11	0.062
ADFI, g/d	11.89 ± 0.11	11.79 ± 0.11	11.97 ± 0.07	0.478
FCR	3.44 ± 0.22	2.92 ± 0.08^*^	3.19 ± 0.09	0081
D21-D28				
ADG, g/d	6.61 ± 0.52	7.66 ± 0.35	6.93 ± 0.29	0.201
ADFI, g/d	19.99 ± 0.82	19.51 ± 0.62	20.27 ± 0.95	0.804
FCR	3.07 ± 0.17	2.56 ± 0.08^**^	2.94 ± 0.16	0.064
D28-D35				
ADG, g/d	9.55 ± 0.61	12.92 ± 0.41^***^	9.62 ± 0.47	0.001
ADFI, g/d	30.09 ± 1.21	29.44 ± 1.58	29.36 ± 1.52	0.927
FCR	3.18 ± 0.16	2.28 ± 0.10^***^	3.08 ± 0.20	0.003

**Table 3:** Effect of MLP on the growth performance of immunosuppressed chicks.

D28-D35	NC	MC	MLP + CTX	MLE + CTX	*p*-value
ADG, g/d	9.55 ± 0.61	5.66 ± 0.42^***^	7.68 ± 1.11^**#**^	7.66 ± 0.59^**#**^	0.015
ADFI, g/d	30.09 ± 1.21	26.77 ± 1.29	27.72 ± 1.52	29.98 ± 0.56	0.168
FCR	3.18 ± 0.16	4.79 ± 0.32^***^	3.87 ± 0.45^**#**^	3.99 ± 0.27	0.021

Data are expressed as mean ± SD. ^*^p < 0.05, ^**^p < 0.01, ^***^p < 0.001 compared with the NC group. ^#^*p* < 0.05, compared with MC.

**Figure 3:**
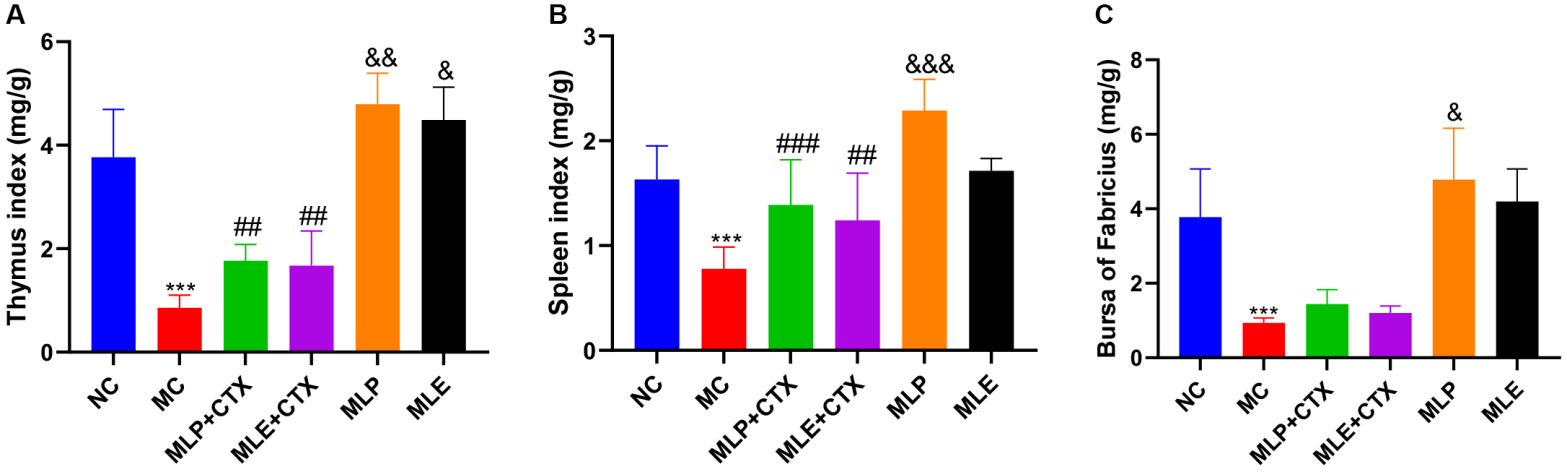
Effect of MLP on immune organ indices in chicks. **(A)** Thymus index, **(B)** Spleen index, **(C)** Bursa index. Compared with the NC group, ^*^p < 0.05, ^**^p < 0.01, ^***^p < 0.001; compared with the MC group, ^#^*p* < 0.05, ^##^*p* < 0.01, ^###^*p* < 0.001; compared with the NC group, ^&^*p* < 0.05, ^&&^*p* < 0.01, ^&&&^*p* < 0.001.

In addition, indexes of spleen, thymus, and BF were observed to evaluate the immune function of chicks; as a result, the MC group showed extremely significant lower immune organ indexes relative to the NC group (*p* < 0.001), suggesting the successful establishment of immunosuppression model. On day 35, the spleen index and the thymus index of MLP + CTX and MLE + CTX groups significantly increased relative to the MC group (*p* < 0.01), while the bursa index was not significantly different, but there was an increasing trend. Meanwhile, the spleen index, the thymus index, and the bursa index of the MLP group significantly increased relative to the NC group (*p* < 0.05). The MLE group was not significantly different from the NC group, with the exception of the thymus index that markedly elevated (*p* < 0.05; Figure 3).

### 3.3 Function of MLP in serum cytokines and immunoglobulins in chicks

**Figure 4:**
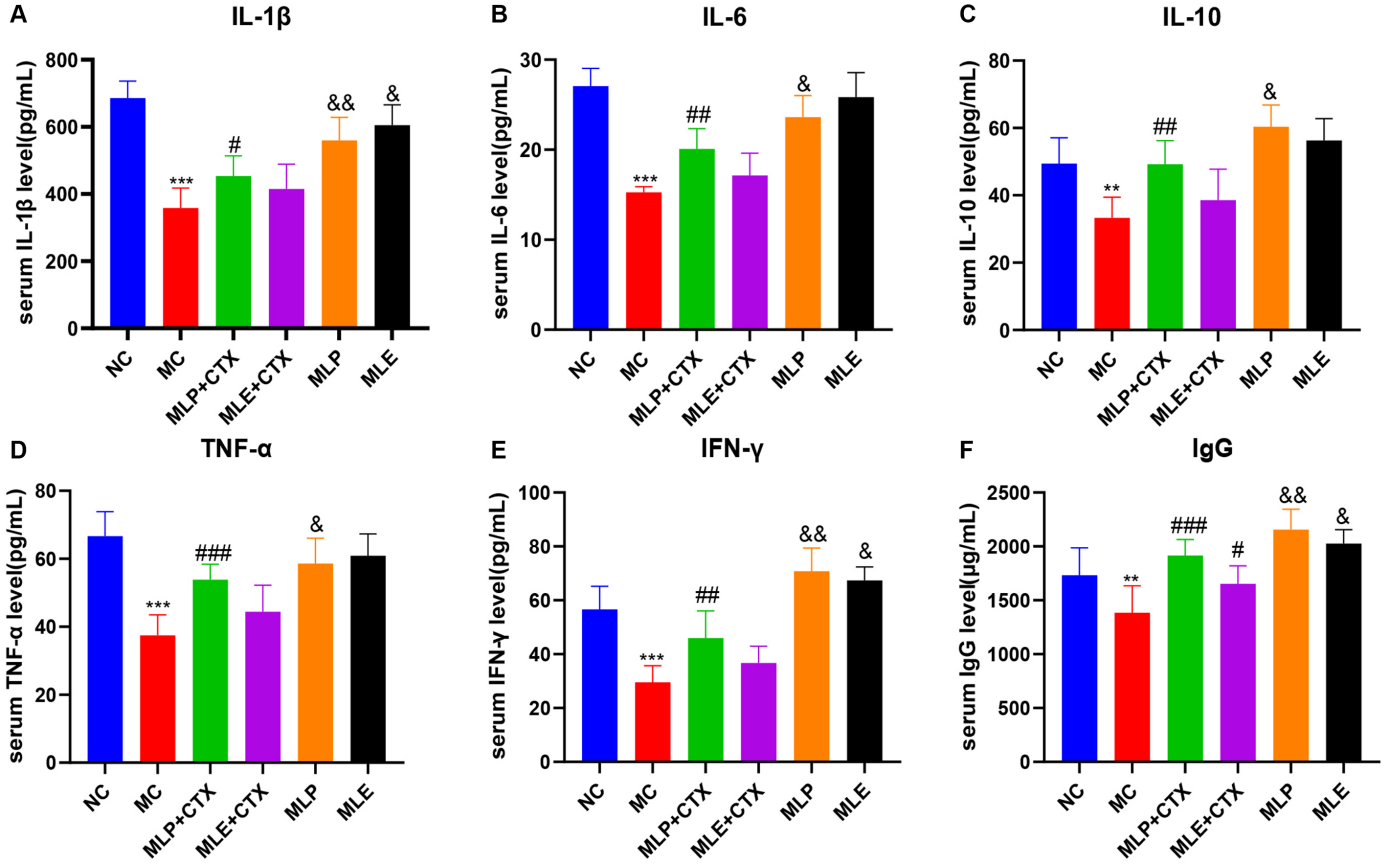
Effect of MLP on cytokines and immunoglobulins in the serum of chicks. **(A)** Serum levels of IL-1β, **(B)** serum levels of IL-6, **(C)** serum levels of IL-10, **(D)** serum levels of TNF-α, **(E)** serum levels of IFN-γ, and **(F)** serum levels of IgG. Compared with the NC group, ^*^p < 0.05, ^**^p < 0.01, ^***^p < 0.001; Compared with the MC group, ^#^*p* < 0.05, ^##^*p* < 0.01, ^###^*p* < 0.001; compared with the NC group, ^&^*p* < 0.05, ^&&^*p* < 0.01, ^&&&^*p* < 0.001.

According to our results regarding the function of MLP in cytokines and immunoglobulins of chick serum, serum IL-1β, IL-6, IL-10, IFN-γ, TNF-α, and IgG levels in the MC group showed extremely significant decreased levels relative to the NC group (*p* < 0.01 or *p* < 0.001), and the above results proved that CTX could inhibit the immune activity of chickens. Meanwhile, relative to the MC group, serum cytokines and IgG levels of MLP + CTX group significantly increased (*p* < 0.05 or *p* < 0.01), but the difference was not significant compared with the MLE + CTX group except for levels of IgG (*p* < 0.05). Surprisingly, the MLP group was significantly different from the NC group, with the MLP group had markedly lower IL-1β, IL-6, and TNF-α levels in serum (*p* < 0.05), but evidently higher IL-10, IFN-γ, and IgG levels (*p* < 0.05), while the MLE group, except for the significant elevation of IgG (*p* < 0.05), there was no significant difference in other indicators compared to the NC group (Figure 4).

### 3.4 Effect of MLP on serum antioxidant enzyme activity in chicks

**Figure 5:**
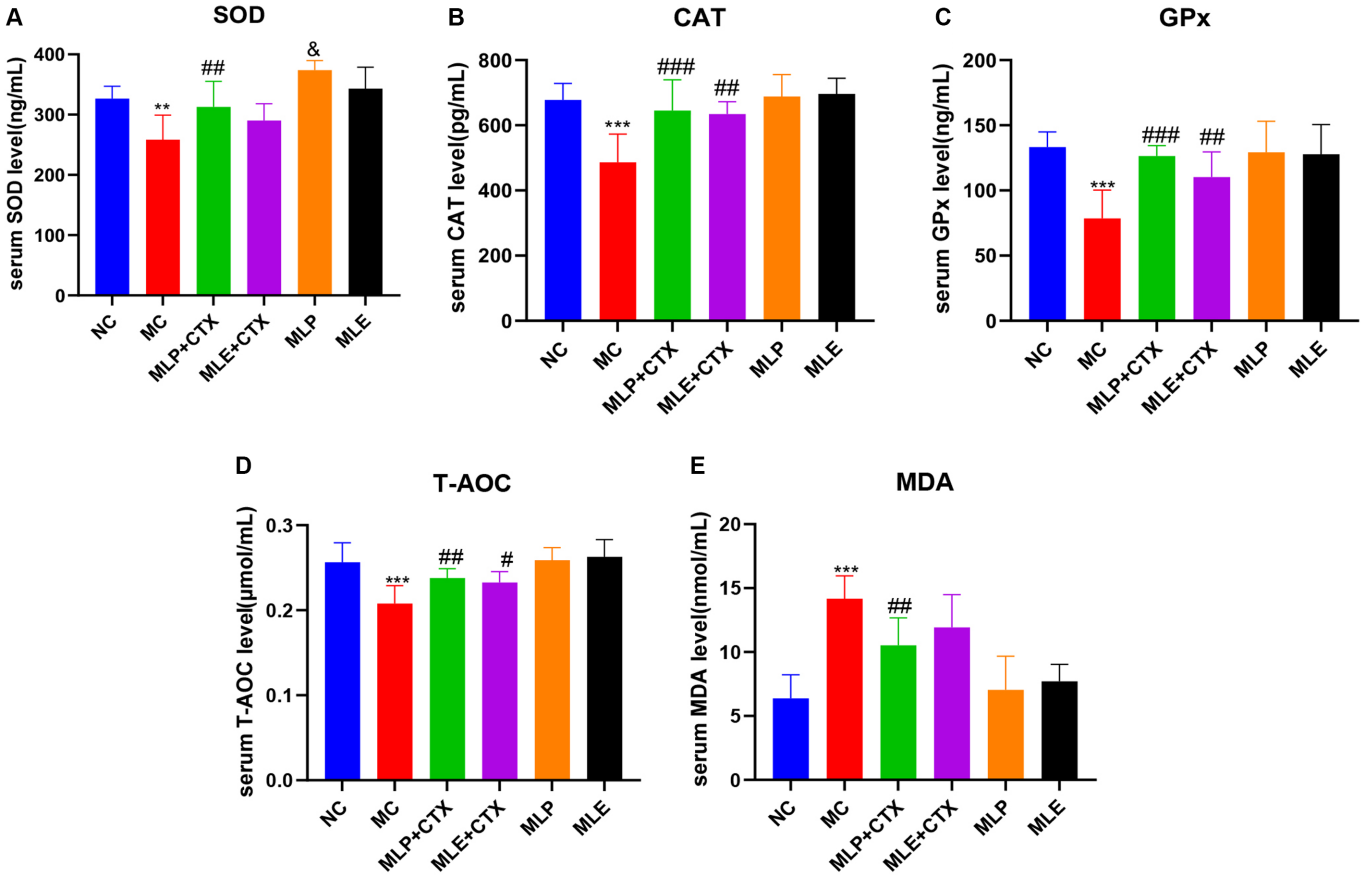
Effect of MLP on antioxidant enzymes in the serum of chicks. **(A)** Serum levels of SOD, **(B)** serum levels of CAT, **(C)** serum levels of GPx, **(D)** serum levels of T-AOC, and **(E)** serum levels of MDA. Compared with the NC group, ^*^p < 0.05, ^**^p < 0.01, ^***^p < 0.001; compared with the MC group, ^#^*p* < 0.05, ^##^*p* < 0.01, ^###^*p* < 0.001; compared with the NC group, ^&^*p* < 0.05, ^&&^*p* < 0.01, ^&&&^*p* < 0.001.

The results of antioxidant enzyme activities in chick serum showed that the MC group exhibited remarkably decreased SOD, CAT, GPx, and T-AOC serum levels relative to the NC group (*p* < 0.01 or *p* < 0.001), while MDA levels were extremely markedly upregulated relative to the NC group (*p* < 0.001), which proved that CTX could also cause oxidative stress in chicks. Meanwhile, relative to the MC group, antioxidant enzymes highly significantly increased (*p* < 0.05 or *p* < 0.01), while MDA content extremely markedly declined (*p* < 0.01) in the MLP + CTX group, while the MLE + CTX group was not significantly different except for CAT, GPx, and T-AOC contents, which remarkably elevated (*p* < 0.05). The MLP and MLE groups were not significantly different from the NC group. It is thus clear that both MLP and MLE prevent oxidative stress resulting from CTX in the body (Figure 5).

### 3.5 Effect of MLP on the pathological damage of chick jejunum

**Figure 6:**
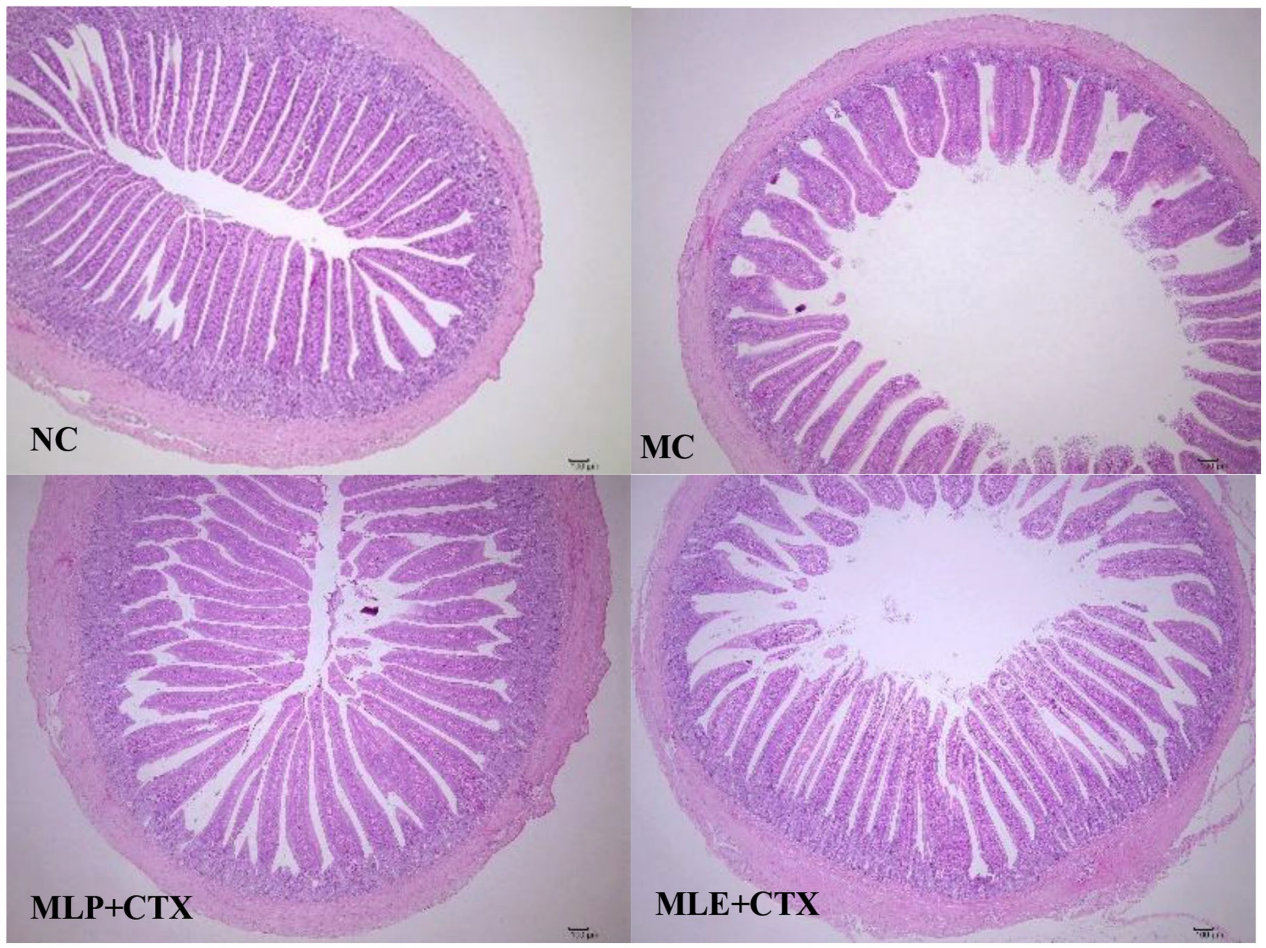
Effect of MLP on the histological structure of the jejunum of the chick.

**Figure 7:**
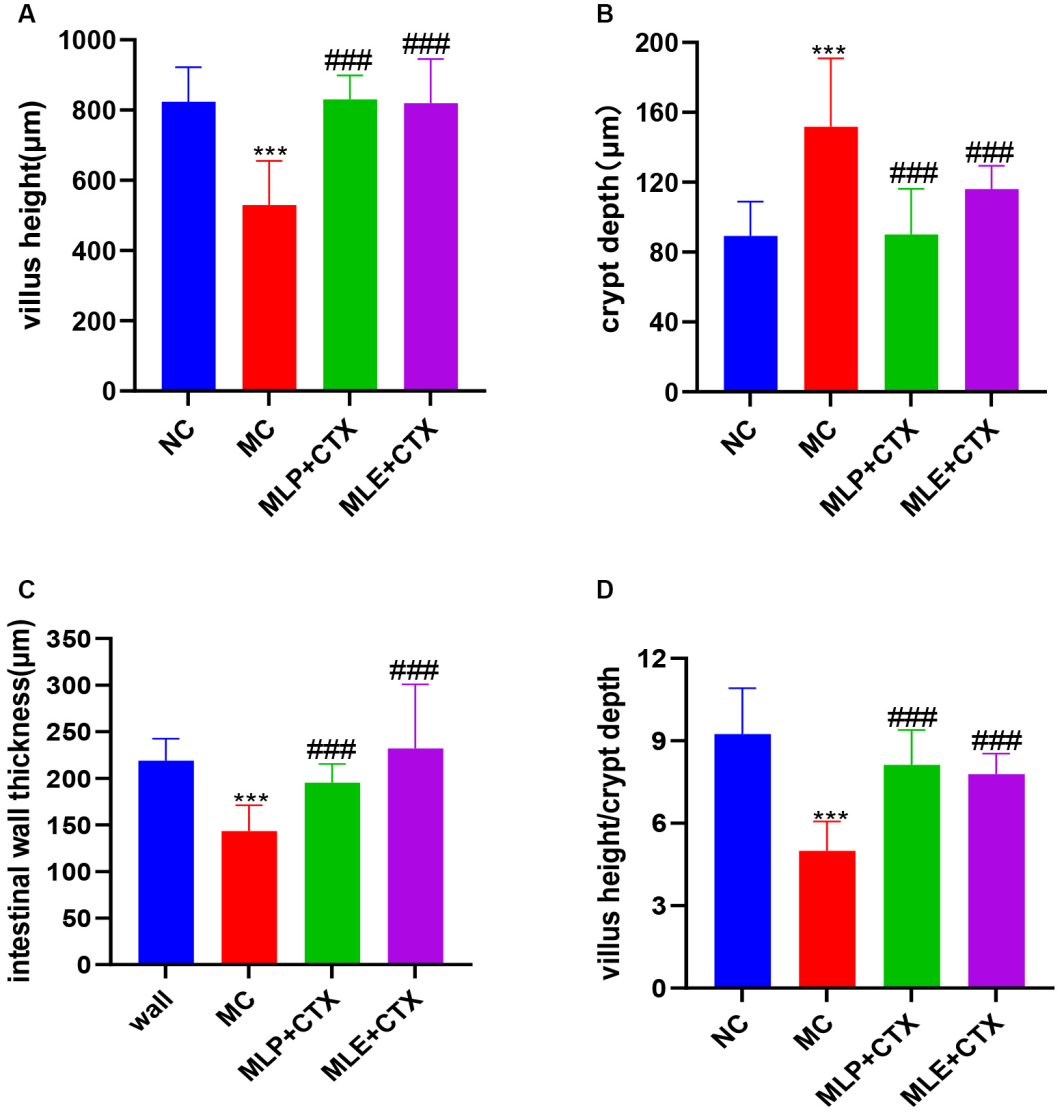
Effect of MLP on the intestinal morphology of chicks. **(A)** Villus height, **(B)** crypt depth, **(C)** intestinal wall thickness, and **(D)** villus height/crypt depth. Compared with the NC group, ^*^p < 0.05, ^**^p < 0.01, ^***^p < 0.001; compared with the MC group, ^#^*p* < 0.05, ^##^*p* < 0.01, ^###^*p* < 0.001.

Based on the HE staining results, the NC group showed complete and orderly intestinal morphology, complete intestinal villi, complete and fine structure, and shallow crypts. The jejunum morphology in chicks of the MC group was severely impaired, with destructed, short and rough intestinal villi, and deeper recess. After MLP treatment, the intestinal status returned to similar to that of the NC group (Figure 6). By measuring the villus height, crypt depth and intestinal wall thickness of the chicks’ jejunum (Figure 7), we can obtain the following conclusions, villi height, villi crypt ratio and intestinal wall thickness of MC group extremely markedly decreased in comparison with NC group (*p* < 0.001) and crypt depth was highly significant higher than NC group (*p* < 0.001), after MLP and MLE administration, villi height, villi crypt ratio and intestinal wall thickness extremely remarkably increased (*p* < 0.001), while crypt depth remarkably decreased (*p* < 0.001) in both MLP and MLE treatment.

### 3.6 Function of MLP in jejunum-associated genes levels within immunosuppressed chicks

**Figure 8:**
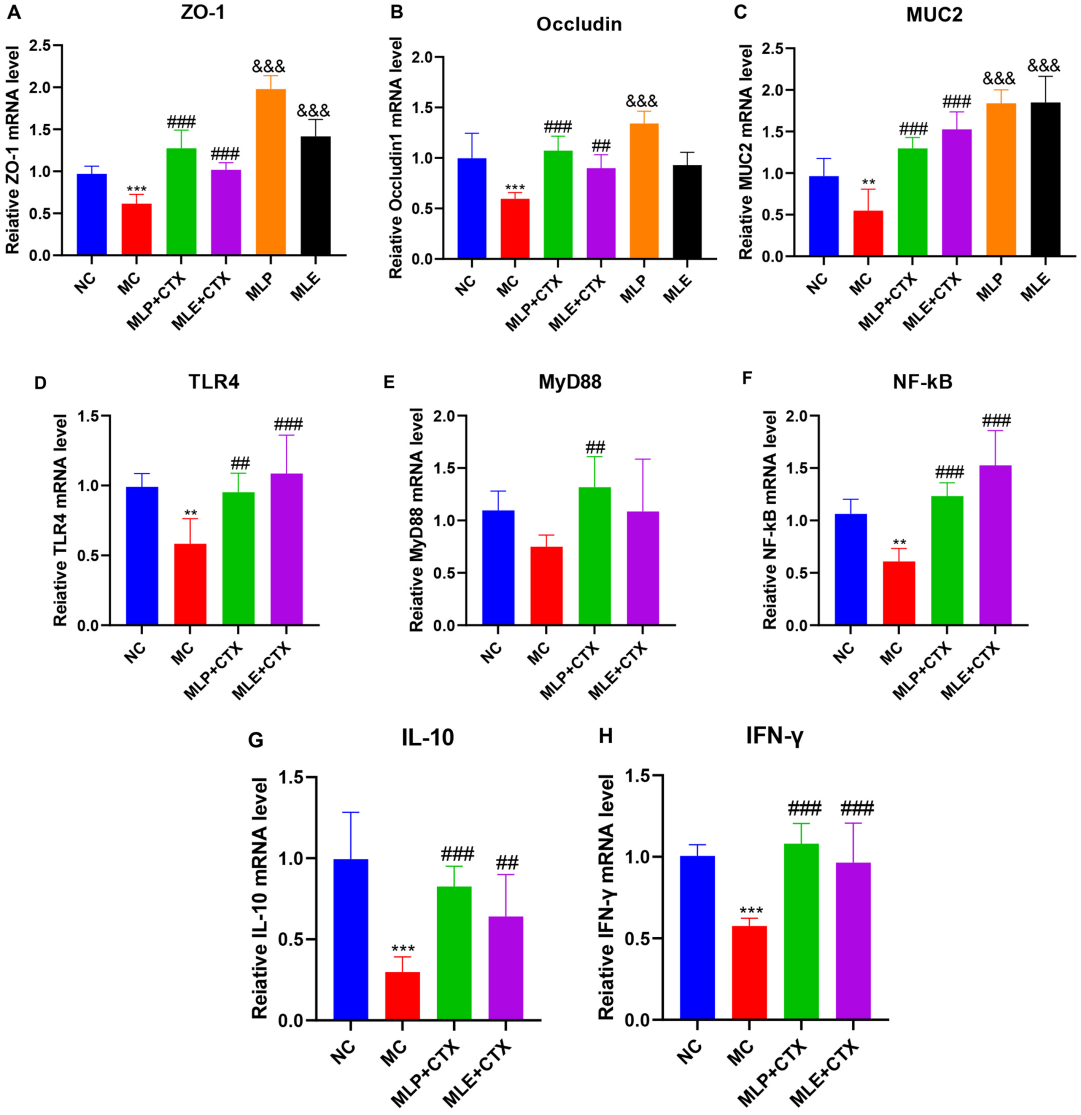
Effect of MLP on the expression of jejunum-related genes. **(A)** Expression of ZO-1, **(B)** expression of Occludin, **(C)** expression of MUC2, **(D)** expression of TLR4, **(E)** expression of MyD88, **(F)** expression of NF-κB, **(G)** expression of IL-10, and **(H)** expression of IFN-γ. Compared with the NC group, ^*^p < 0.05, ^**^p < 0.01, ^***^p < 0.001; compared with the MC group, ^#^*p* < 0.05, ^##^*p* < 0.01, ^###^*p* < 0.001.

As exhibited by jejunal Occludin, ZO-1, MUC2, TLR4, MyD88, NF-κB, IL-10, and IFN-γ gene levels, the MC group showed significantly decreased expression of Occludin, ZO-1, and MUC2 relative to the NC group (*p* < 0.001, *p* < 0.001, *p* < 0.01), while the expressions in both the MLP + CTX and MLE + CTX groups significantly increased relative to the MC group (*p* < 0.001); meanwhile, the expression of Occludin, ZO-1, and MUC2 of the MLP group significantly increased relative to the NC group (*p* < 0.001), while only ZO-1 and MUC2 levels of the MLE group significantly increased compared with the NC group (*p* < 0.001; Figure 8).

As for mRNA levels, TLR4 and NF-κB levels of the MC group were both extremely remarkably downregulated in comparison with the NC group (*p* < 0.01), MyD88 expression was second (*p* > 0.05), and the difference was not significant, while TLR4, MyD88, and NF-κB of MLP + CTX group were all significantly increased in comparison with the MC group (*p* < 0.001, *p* < 0.01, *p* < 0.001), TLR4 and NF-κB of the MLE + CTX group were significantly upregulated (*p* < 0.001), while MyD88 expression was not significantly different in the MLE + CTX group (*p* > 0.05).

IL-10 and IFN-γ gene expression results in jejunum showed that IL-10 and IFN-γ levels of the MC group were highly significantly lower than NC group (*p* < 0.001), and IL-10 and IFN-γ levels of the MLP + CTX group were significantly higher than MC group (*p* < 0.001) and nearly returned to the level in NC group, meanwhile, the expressions of IL-10 and IFN-γ in MLE+CTX group were significantly higher than those in MC group (*p* < 0.01). This conforms to serum measurements.

### 3.7 Function of MLP in the intestinal flora within immunosuppressed chicks

Cecal feces in D35 chicks were collected to conduct high-throughput 16S rDNA gene sequencing analysis. First, rarefaction curve analysis revealed the reasonability and reliability of sample number and sequencing depth (Supplementary Figure S1). Alpha-diversities were analyzed to determine microbial community abundance and diversity. The results below were summarized by various indexes for statistical analyses, including the sobs, chao, Shannon, ace, and simpson. Of them, the sobs, chao and ace indexes reflected community richness; as a result, CTX decreased microbial richness within cecal samples in chicks, while MLP and MLE alleviated the decrease in richness caused by CTX (*p* > 0.05), implying that MLP and MLE elevated intestinal microbial number; second, the shannon and simpson indices indicated microbial community diversity, and our results showed that MLP and MLE also elevated microbial community diversity after treatment (Table 4).

**Table 4:** α-Diversity indices of gut microbiota in each group.

Groups	NC	MC	MLP + CTX	MLE + CTX	*p*-value
sobs	456.8 ± 57.89	401.7 ± 149.1	413.7 ± 82.42	427.2 ± 54.99	0.8479
chao	513.9 ± 50.31	472.3 ± 172.3	471.8 ± 98.03	492.3 ± 55.58	0.7867
shannon	3.613 ± 0.4203	3.326 ± 0.882	3.691 ± 0.278	3.699 ± 0.2938	0.9204
ace	528.2 ± 53.12	464.5 ± 177.3	472 ± 100.2	498.4 ± 54.88	0.6823
simpson	0.0827 ± 0.037	0.1215 ± 0.1093	0.0652 ± 0.013	0.0644 ± 0.022	0.6106

Data are expressed as mean ± SD. ^*^p < 0.05, ^**^p < 0.01, ^***^p < 0.001 compared with the NC group. ^#^*p* < 0.05, compared with MC.

**Figure 9:**
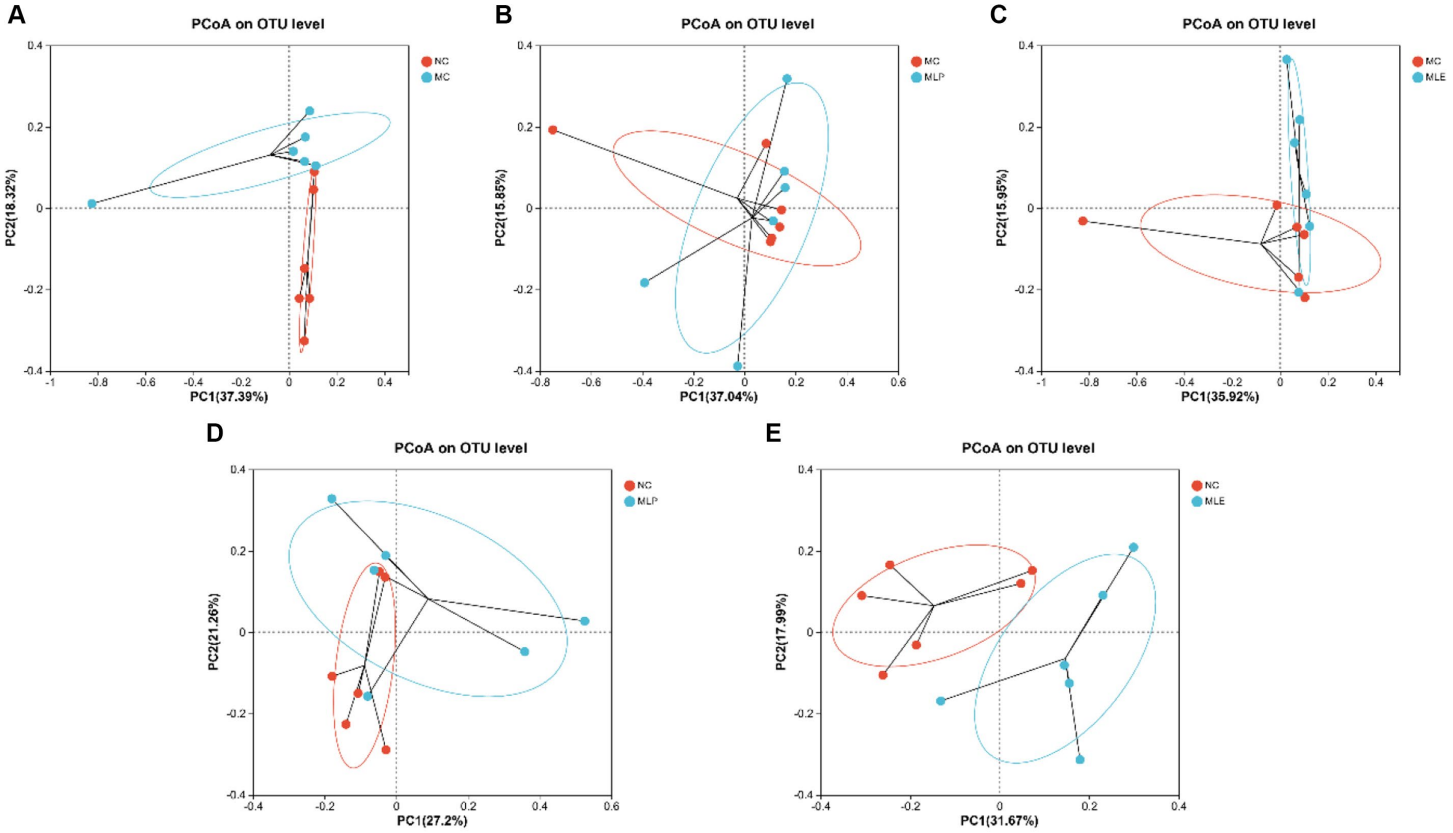
PCoA of intestinal microorganisms in chicks **(A)** NCvsMC, **(B)** MCvsMLP, **(C)** MCvsMLE, **(D)** NCvsMLP, and **(E)** NCvsMLE.

Additionally, beta-diversities were analyzed to examine similarities and differences among sample communities. There is a more obvious separation in the NC group compared with the MC group, indicating a large difference between them; also observing the relationship between the MLP and MLE groups and the NC group, it can be found that the MLP group is closer to the NC group relative to the MLE group (Figure 9).

**Figure 10:**
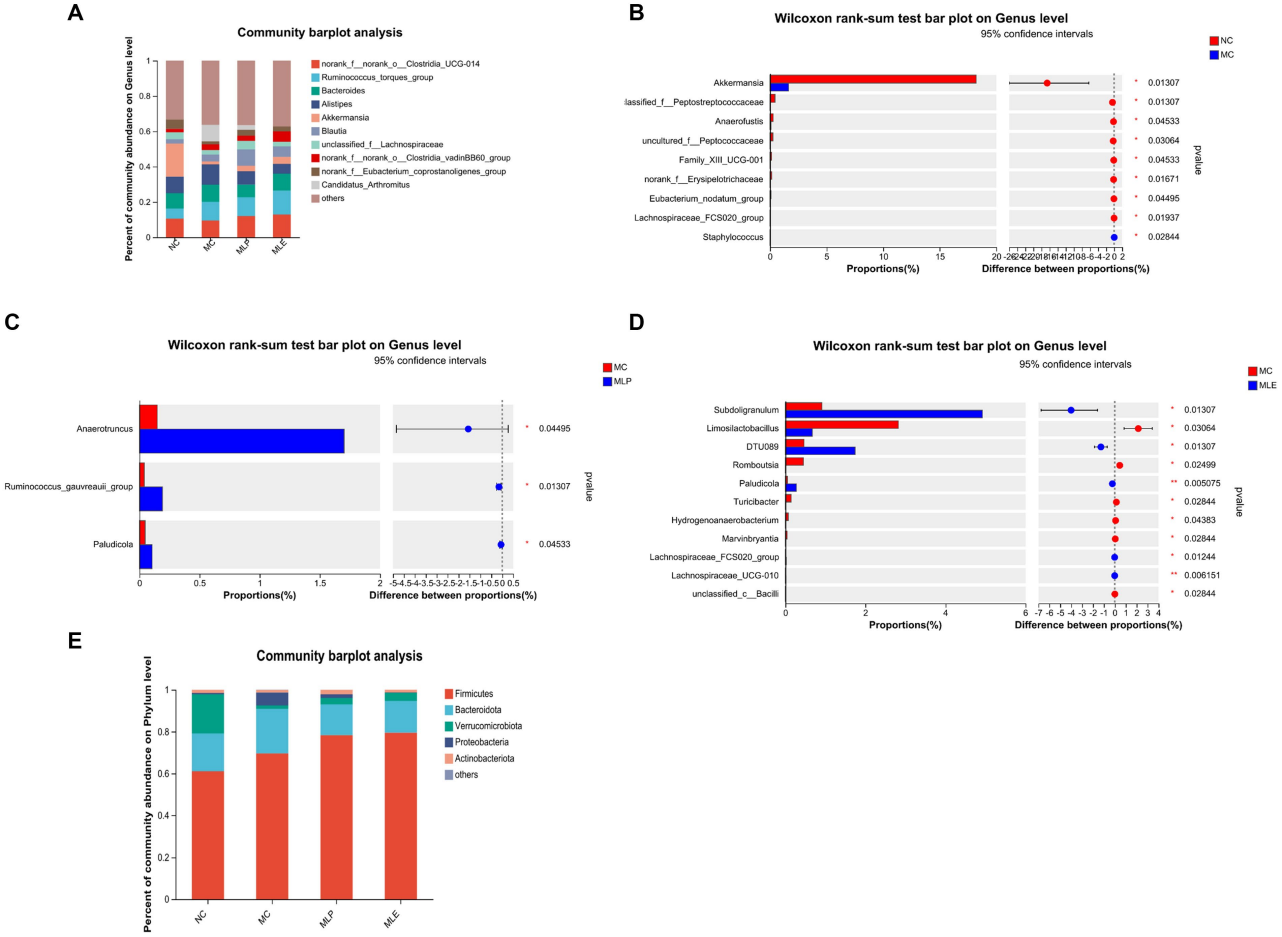
Composition of the intestinal flora of chicks at the genus level and their significantly different species at the genus level. **(A)** Composition of gut microbial genus levels in chicks, **(B)** NC versus MC, **(C)** MC versus MLP, and **(D)** MC versus MLE. The right of the bar is the *p*-value; ^*^p < 0.05, ^**^p < 0.01. **(E)** Composition of each group at the Phylum level.

The composition of the intestinal flora of the chicks was analyzed in terms of phylum. As a result, Firmicutes and Bacteroidetes are the two most dominant phyla in microflora. Relative to the MC group, the MLP and MLE groups tended more toward the NC group, with some recovery of several phylum levels. Further analysis revealed that both the MLP and MLE groups increased the proportion of Firmicutes, Verrucomicrobiota, and Actinobacteriota (*p* > 0.05) and reduced that of Bacteroidota and Proteobacteria (*p* > 0.05) in comparison with the MC group (Figure 10E).

Chick intestinal flora were compared at a genus level; as a result, after CTX treatment, chick intestinal flora of Akkermansia, unclassified_f_Peptostreptococcaceae, Anaerofustis, uncultured_f_Peptococcaceae, Family_XIII_UCG-001, norank_f_Erysipelotrichaceae, Eubacterium_nodatum_group, and Lachnospiraceae_FCS020_group, eight bacterial genera, markedly decreased (*p* < 0.05), while Staphylococcus bacterial counts markedly increased (*p* < 0.05). Compared with the MC group: after MLP treatment, the intestinal proportions of *Anaerotruncus*, Ruminococcus_gauvreauii_group and Paludicola significantly elevated in the MLP group (*p* < 0.05); following MLE treatment, Subdoligranulum, DTU089, Paludicola, Lachnospiraceae_FCS020_group, and Lachnospiraceae_UCG-010 were significantly higher (*p* < 0.05), and Limosilactobacillus, Romboutsia, Turicibacter, and Hydrogenoanaerobacterium, Marvinbryantia, and unclassified_c_Bacilli were significantly lower (*p* < 0.05). In conclusion, mulberry leaf polysaccharides and aqueous extracts can improve the intestinal flora disorder caused by CTX to some extent (Figures 10A–D).

**Figure 11:**
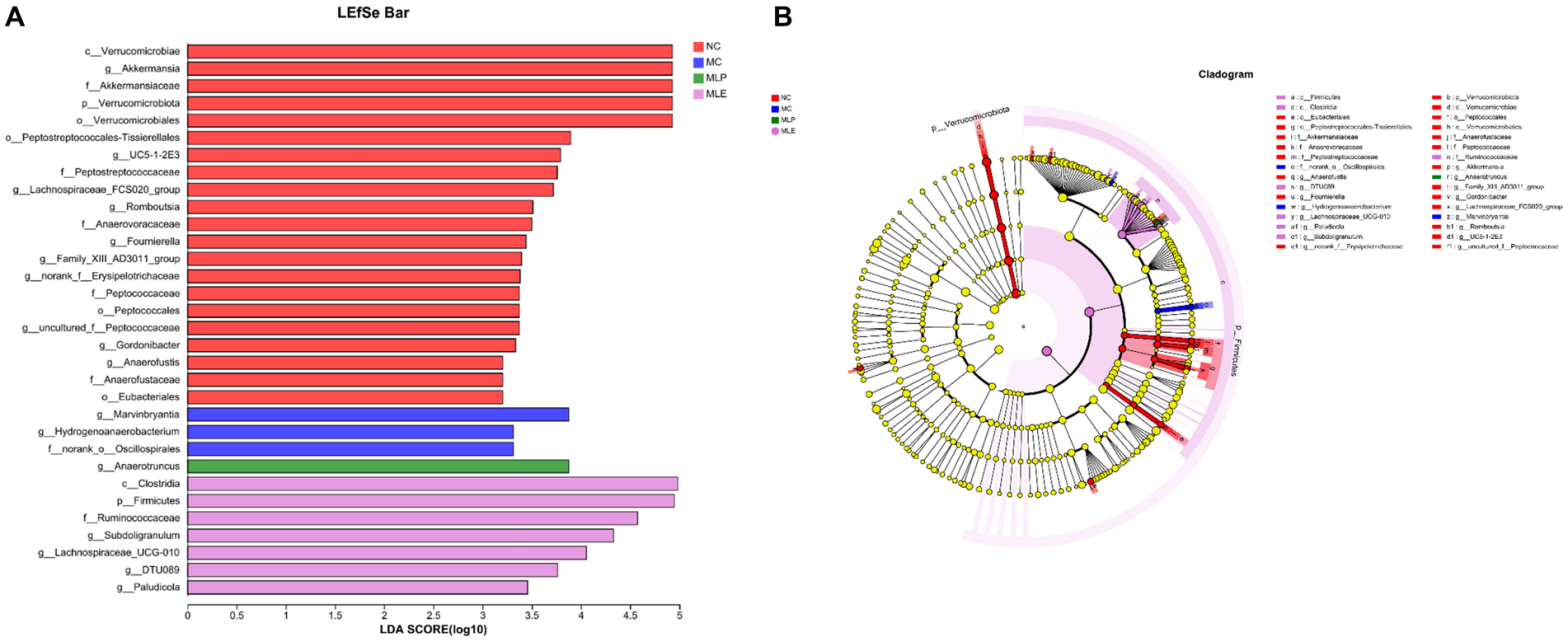
Difference in dominant microorganisms among each group via cladogram and distribution histogram based on LDA. **(A)** Histogram of LDA distribution based on LEfSe analysis of classification information and **(B)** evolutionary branching diagram of LEfSe analysis based on classification information.

As shown in Figure 11, in the NC group c_Verrucomicrobiae, g_Akkermansia, f_Akkermansiaceae, p_Verrucomicrobiota, o_Verrucomicrobiales, and Peptostreptococcales-Tissierellales have increased LDA scores, indicating their higher OUT of the NC group; for the MC group, the high LDA scores are for g_Marvinbryantia, g_Hydrogenoanaerobacterium, and f_norank_o_Oscillospirales These three species indicate that these three species are more susceptible to CTX stimulation; in terms of feeding MLP, g_Anaerotruncus has the highest score; in terms of feeding MLE, c_Clostridia, p_Firmicutes, f_Ruminococcaceae, g_Subdoligranulum, g_Lachnospiraceae_UCG-010, g_DTU089, and g_Paludicola, seven species had higher scores. It indicates that MLP can mitigate CTX-mediated immunosuppression through elevating the intestinal abundance of *Anaerotruncus*.

## 4 Discussion

Modern industrial poultry farming is very large, so the growth performance of poultry is the fundamental guarantee of the benefits generated by the farming industry. It has been shown that a variety of polysaccharides have the function of enhancing the growth performance of poultry; for example, adding LBP to the diet can enhance growth performance, digestive enzyme activity, and immunity of broilers (Long et al., 2020). In addition, we are examining the effect of both MLP and MLE as a whole for feed additives on chicks and therefore take a common dose of 0.25%. Moreover, the selection of the 0.25% addition amount is determined by the results of the previous preliminary experiment, and the methods and results of the preliminary experiment have been supplemented in the Supplementary material. In this experiment, we first verified whether MLP can promote growth performance in chickens. According to the results, adding 0.25% MLP to the diet could significantly improve the ADF of chicks in all periods, but it did not significantly affect AFD in chickens. In terms of feed conversion ratio, MLP could significantly reduce the feed-to-meat ratio of chicks in all periods. CTX injections in chicks resulted in lower average daily weight gain (Cui et al., 2022) and growth performance (Liu et al., 2021). Dietary patterns not only affect weight, but also bone density (Chen et al., 2015). In the present study, growth performance in chicks of the MC group decreased dramatically compared with the NC group, mainly in terms of ADG and ADFI, while the addition of 0.25% MLP in the feed significantly increased the ADG and decreased the FCR of chicks, which shows that MLP promotes growth in chicks in the immunosuppressed state of chicks.

Immune activity is often considered one of the most important defenses against bacterial–viral infections, cancer invasion, or inflammation (Zhu et al., 2016; Li et al., 2019; Qu et al., 2022). The central immune organs include the bursa and thymus, which have a decisive and dominant effect on peripheral lymphoid organ development (Shirani et al., 2015). In some studies, certain natural plant-derived polysaccharide components have the ability to enhance the immune organ index in chickens (Fan et al., 2013; Cui et al., 2022). We found experimentally that CTX extremely markedly reduced the immune organ index in chickens, indicating that the model we established was successful. Meanwhile, the immune organ damage was alleviated by adding MLP and MLE to the feed, but MLE was not as effective as MLP, indicating that MLP promotes immune function in chickens through stimulation of their immune organs.

Cytokines are highly active multi-kinetic protein-peptide molecules produced via immune cells and other related cells, which mainly mediate and regulate the immune response and inflammatory reaction and are involved in tissue repair, among others (Schoenaker et al., 2018). According to the previous articles, some plant-derived polysaccharides mitigate CTX-mediated immunosuppression through immune cell activation, thus promoting cytokine secretion (Zhou et al., 2018; Yang et al., 2021; Zhou et al., 2022). In this study, MLP modulates immunosuppression of chicks through elevating these six cytokine levels, conforming to prior results. Additionally, we examined cytokine secretion in the group without CTX injection, and interestingly, MLP reduced IL-1β, IL-6, and TNF-α secretion compared with the NC group, but those in the MC group significantly increased; it elevated IL-10, IFN-γ, and IgG secretion. The possible reason is that MLP can also regulate the secretion of serum cytokines in chicks to enhance the immunity and anti-inflammatory ability of the organism in the absence of immunosuppression (Zhang et al., 2018). Combining these findings, MLP can enhance cytokine production to alleviate immunosuppression caused by CTX to regulate the systemic immune response.

CTX is capable of causing oxidative stress in the body, after oxidative stress, the antioxidant defense system of the body is disrupted (Has et al., 2019; Zhu et al., 2022). Exogenous dietary additives and ingredients may affect growth performance and antioxidation in animals (Ma et al., 2021; Chen JY et al., 2022; Chen FM et al., 2022). It has been demonstrated that Amaranthus polysaccharides can alleviate CTX-induced oxidative stress in mice (Niu et al., 2020) and enhance the antioxidant enzyme activities of mice in an immunosuppressed state. It has been demonstrated that Mulberry leaf polysaccharides have strong *in vitro* antioxidant effects (Yuan et al., 2015; Zhang et al., 2016), so this study analyzed how MLP affected CTX-mediated oxidative stress; as a result, antioxidant enzyme activities in serum were remarkably decreased, while MDA content evidently increased in the MC group, while antioxidant enzyme levels within the serum were higher and MDA level was significantly lower after our MLP treatment, our findings are consistent with previous studies showing that polysaccharides boost antioxidant capacity.

The intestinal mucosal barrier generally refers to the normal intestine having and perfect functional isolation zone that separates the interior of the intestine from the internal environment of the organism, preventing the invasion of pathogenic bacteria, toxic or carcinogenic substances, keeping the internal environment of the organism stable (Bai et al., 2020; Sha et al., 2021) and enabling the maintenance of the collective normal life activities. Dietary nutrients can affect animal small intestine morphology (Chen et al., 2019). An imbalance of gut microbiota–other factor interaction may break the intestinal mucosal homeostasis (Luo et al., 2022). The intestinal epithelial barrier is the first-line defense between the lumen and the host, and if damaged, it may lead to intestinal disorders like inflammation (Wu et al., 2020). According to our results, the intestinal mucosal integrity of chicks was disrupted after CTX injection and the long villi became disorganized and flaccid. After MLP intervention, the morphology of the intestine was changed and the pathological damage was attenuated, indicating that MLP could improve CTX-induced intestinal damage. The intestinal villi and crypt account for critical indexes for describing intestinal function and morphology. This shows that both MLP and MLE significantly affect CTX-mediated intestinal injury, and both can protect intestinal mucosal function by regulating jejunal injury and restoring intestinal absorption and digestive abilities, conforming to previous findings by Cui et al. (2022) and Zhou et al. (2021).

Intestinal tight junction proteins are crucial for keeping intestinal integrity and permeability, with the main function of closing the epithelial cell gap (Schoultz and Keita, 2019). MUC2 represents the intestinal-type mucin produced via cupped cells that forms intestinal mucus while resisting bacterial destruction (Cai et al., 2021). Additionally, defective epithelial MyD88 signaling targeting has been reported to lead to increased mucus-related bacterial number and decreased MUC2 levels (Frantz et al., 2012). Polysaccharides are found to restore CTX into intestinal mucosal injury through regulating intestinal tight junction proteins, for example, Ganoderma lucidum polysaccharides (Ying et al., 2020), *Cordyceps polysaccharides* (Ying et al., 2020), and Oxaliplatin polysaccharides (Chen et al., 2021) reversed intestinal damage by upregulating intestinal tight junction proteins. In the present study, we showed that both MLP and MLE treatment could upregulate MUC2, Occludin, and ZO-1 proteins within the jejunum in chickens after CTX injection, indicating that MLP and MLE can be important for CTX-mediated intestinal mucosal injury. Interestingly, MLP and MLE also remarkably upregulated MUC2, Occludin, and ZO-1 in the group without CTX injection, Occludin, and ZO-1 expression, revealing that MLP and MLE can increase intestinal permeability and digestive absorption through upregulation of MUC2, Occludin and ZO-1 expression, which is related to relationship with growth performance that we previously examined. In addition, we examined IL-10 and IFN-γ levels within chicken jejunum, and our results were consistent with those of our previously examined serum, suggesting that MLP also promotes immune cytokine generation via the intestinal immune system through upregulating IL-10 and IFN-γ expression. Therefore, these cytokines can migrate to the spleen and peripheral lymph nodes, thereby triggering systemic immunity.

The Toll-like receptor (TLR) family has received wide attention as the possible regulatory factors and controllers for immunity by recognizing pathogen-associated molecular patterns (Zuany-Amorim et al., 2002). TLRs are crucial for immune cell regulation, proliferation, and survival, which exert a critical effect on intestinal immunity (Dong et al., 2019). TLRs and glycosyl ligands can bind to and signal MyD88, thereby activating the NF-B pathway while inducing cytokine production, finally stimulating immunity (Zhu et al., 2013; Zhou et al., 2020). It has been demonstrated that the ability of polysaccharides to modulate immunity is linked to TLR4-mediated signaling pathways and that Caulis Spatholobi polysaccharide promotes the increased TLR4, MyD88, and NF-κB levels, key immune signals in the intestine, restoring immune performance (Cui et al., 2022), and it has also been shown that Millettia Speciosa Champ polysaccharide modulate immune performance in mice through TLR4-induced MyD88 pathway activation (Chen et al., 2021). In this study, CTX caused decreased TLR4, MyD88, and NF-κB levels after CTX injection, and we observed that MLP could significantly upregulate TLR4, MyD88, and NF-κB of the MC group. In conclusion: MLP can upregulate intestinal tight junction protein, upregulate TLR4, MyD88, and NF-κB gene expression, and regulate intestinal immunity, thereby protecting the intestinal mucosal barrier while promoting growth.

With the continuous advancement of sequencing technology, we have the opportunity to conduct in-depth research on how dietary alterations affect animal intestinal microbiomes (He et al., 2020; Wang et al., 2022; Chen et al., 2023; Liu et al., 2023). The intestinal microbial milieu has a critical effect on the organism, mainly on inhibiting pathogenic microbial colonization, keeping intestinal epithelial integrity, regulating host organism immunity, and modulating innate immunity. It has been shown that natural plant-derived polysaccharide active ingredients promote immunity through the continuous stimulation of host immunity, thus stimulating changes in the flora to activate the immune response (Zhou et al., 2021); for example, Cordyceps polysaccharides elevate probiotic bacterial abundances in the intestine and decrease pathogenic bacterial production, thus alleviating the side effects associated with CTX (Frantz et al., 2012). First, higher bacterial diversity in the gut usually implies a more stable microbial community in the ‘gut of the organism, with a richer microbial community, thus maintaining the health status of the animal organism by suppressing pathogen colonization and maintaining immune homeostasis (Elson and Cong, 2012). In the present study, microbial abundance and diversity in the cecum of chicks were reduced after CTX injection, which is consistent with previous studies (Zheng et al., 2022), and were alleviated after MLP and MLE feeding interventions, indicating that dietary MLP and MLE efficiently avoid the reduced bacterial abundance and diversity resulting from CTX. The β-diversity analysis showed that CTX injection significantly altered the microbial community structure, while the dietary MLP resulted in a more convergent microbial community structure toward the NC group, suggesting that MLP can regulate the gut microbial community toward normal levels. To verify this hypothesis, we also examined the alterations of gut microbial composition and specific taxa.

Changing dietary formulas may cause changes in animal gut microbial composition and affect digestion activity (Ji et al., 2019; Wang et al., 2022). We analyzed the MLP and MLE to comparatively analyze intestinal flora at diverse taxonomic levels (phylum and genus levels). At the phylum level, Firmicutes and Bacteroidetes represent phyla with the highest abundances, which use polysaccharides to produce carbohydrate-active enzymes. According to this study, Bacteroidota had an elevated relative abundance of the MC group relative to the NC group, which indicates that Bacteroidetes may use CTX to produce immunosuppression in chicks (Nawaz et al., 2018). In contrast, after feeding MLP and MLE, the Firmicutes, Verrucomicrobiota, and Actinobacteriota percentages increased, whereas Bacteroidota and Proteobacteria percentage decreased in comparison with the MC group, and it has been shown that Firmicutes enhances protein digestion and absorption of animal organism (Janczyk et al., 2009) and can break down the polysaccharide components of plant species, while Verrucomicrobiota can effectively enhance the intestinal immunity in animal organisms (Kong et al., 2020). MLP and MLE enhance the immunity of the intestine of an organism mainly by increasing intestinal Firmicutes and Verrucomicrobiota percentages.

We also analyzed differences in intestinal flora at a genus levela. First, after CTX treatment, altogether eight bacterial genera significantly decreased and one bacterial genus significantly increased, and Staphylococcus apparently elevated of the MC group, suggesting that after CTX administration to the chicks, the immunity of the chicks is reduced, allowing a large invasion of Staphylococcus, which can cause the organism to trigger and promote an inflammatory response. The major bacterial genus Akkermansia was significantly reduced, Akkermansia is a beneficial intestinal bacterium that effectively reduces the level of inflammation in the intestine and gradually decreases with the progression of enteritis (Pope et al., 2012). After MLP feeding, *Anaerotruncus* spp. increased significantly, and it has been shown that *Anaerotruncus* promotes the production of butyric acid (Derrien et al., 2011); butyric acid mainly functions in maintaining intestinal cell stability and protecting them from external pathogens; therefore, the increased abundance of *Anaerotruncus* causes the organism to produce more butyric acid to protect the intestinal cells. This was followed by a significant rise in the genus Ruminococcus_gauvreauii_group. It has been demonstrated that Ruminococcus has highly complex and specific enzymes that have the ability to digest a variety of enzymes, such as cellulase and amylase, and MLP increased the abundance of Ruminococcus, suggesting that MLP enhances digestive enzyme activities through elevating Ruminococcus abundance, which may be associated with capacity of MLP of increasing chick growth performance. First, the MLE group compared with the MC group Subdoligranulum Paludicola was significantly enriched and it has been shown that this species of bacteria produces butyrate, which can help reduce the risk of colon cancer in the organism through promoting intestinal barrier and organism immunity (Zhao et al., 2021). Two genera Lachnospiraceae_FCS020_group, Lachnospiraceae_UCG-010 belong to the genus Trichoderma, represent possible beneficial bacteria related to the metabolism of various carbohydrates, and fermentation can promote acetic acid and butyric acid generation, which provides a major energy source of the host (Suen et al., 2011). Secondly, compared with the MC group, the abundance of six bacteria in the MLE group significantly decreased, while the abundance of the pathogenic bacterium Romboutsia significantly decreased after feeding with MLE, which is consistent with previous research results (Le Roy et al., 2022). In conclusion, MLP and MLE can modulate chick intestinal flora by regulating certain probiotic bacterial enrichment and reducing certain harmful bacterial levels, thus regulating immunity.

## 5 Conclusion

In conclusion, MLP can restore the growth performance of chicks, protect immune organs, promote production of serum cytokines and immunoglobulins, and modulate humoral immunity of the body. MLP contributes to enhancing the antioxidant capacity of chicks, repairing injured intestinal mucosa, enhancing tight junction protein levels, activating intestinal immune-related pathways, and regulating intestinal immunity. In addition, MLP can increase the diversity of microbial communities while regulating microbial community structure.

## Data availability statement

The original contributions presented in the study are included in the article/Supplementary material, further inquiries can be directed to the corresponding authors.

## Ethics statement

The animal study was approved by Committee on Ethics of Animal Experiments of Guangxi University. The study was conducted in accordance with the local legislation and institutional requirements.

## Author contributions

MC: Formal analysis, Investigation, Methodology, Software, Validation, Visualization, Writing – original draft. YS: Investigation, Validation, Writing – review & editing. YC: Investigation, Writing – review & editing. HH: Software, Supervision, Writing – review & editing. SL: Investigation, Writing – review & editing. YX: Investigation, Writing – review & editing. LH: Investigation, Writing – review & editing. SH: Investigation, Writing – review & editing. YL: Investigation, Writing – review & editing. FC: Investigation, Writing – review & editing. JL: Supervision, Writing – review & editing. HS: Conceptualization, Funding acquisition, Methodology, Project administration, Resources, Writing – review & editing, Formal analysis.
