# Supplementary material for: Mulberry leaf polysaccharide improves cyclophosphamide-induced growth inhibition and intestinal damage in chicks by modulating intestinal flora, enhancing immune regulation and antioxidant capacity
Source: Front Microbiol. 2024 Mar 21;15:1382639. doi: 10.3389/fmicb.2024.1382639 (PMC10991686; doi:10.3389/fmicb.2024.1382639)
Supplement: Supplementary file 1 [file Table_1.DOCX]

Supplementary Material


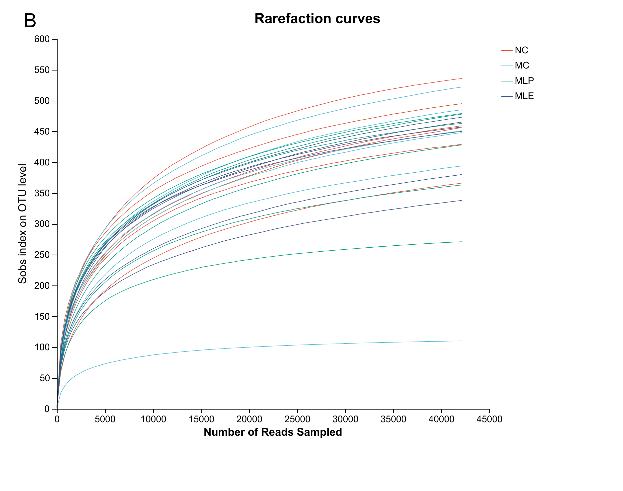

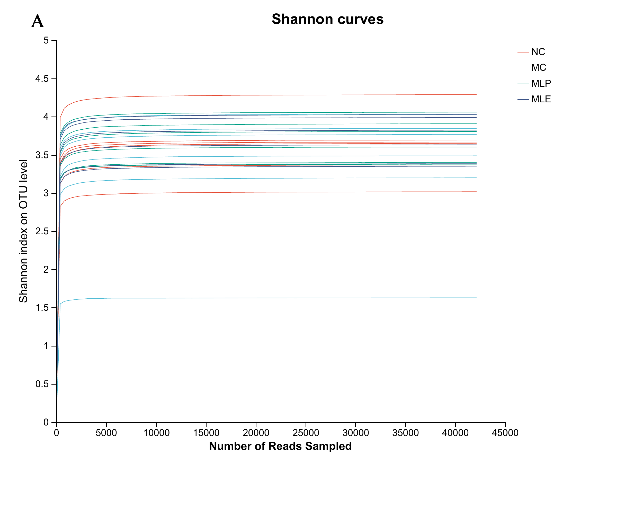


**Supplementary Figures 1.** Rarefaction curve. (A) Shannon Index; (B) Sobs Index.

**Supplementary Table 1**. Primers used in Quantitative Real-time PCR

| **Target gene** | **Primer sequence(5’-3’)** |
| --- | --- |
| β-actin | F：TTGTTGACAATGGCTCCGGT  R：TCTGGGCTTCATCACCAACG |
| TLR4 | F：TTCAGAACGGACTCTTGAGTGG  R：CAACCGAATAGTGGTGACGTTG |
| MyD88 | F：CCGTATGGGCATGGAACAGA  R：CTGGCAAGACATCCCGATCA |
| NF-κB | F：CTACTGATTGCTGCTGGAGTTG  R：CTGCTATGTGAAGAGGCGTTGT |
| ZO-1 | F：CCACTGCCTACACCACCATCTC  R：CGTGTCACTGGGGTCCTTCAT |
| Occludin | F：CGCAGATGTCCAGCGGTTACT  R：CAGAGCAGGATGACGATGAGGAA |
| Muc-2 | F：AATGCTGAGTTCTTGCCTAA  R：TGTTGCAGTTCATATCCTGGT |
| IL-10 | F：GCGCTTCTACACAGATGAGGT  R：CGAACGTCTCCTTGATCTGC |
| IFN-γ | F：ATGTAGCTGAGGGTGGACCT  R：ACGCCATCAGGAAGGTTGTT |

**Methods and results of preparatory experiments**

**Methods**

| **Items** | **NC** | **0.125%** | **0.25%** | ***p*-value** |
| --- | --- | --- | --- | --- |
| **D21-D28** |  |  |  |  |
| ADG, g/d | 6.14±0.14 | 6.14±0.16 | 6.82±0.38 | 0.153 |
| ADFI, g/d | 19.44±0.17 | 18.54±0.34**^*^** | 19.37±0.16 | 0.047 |
| FCR | 3.17±0.06 | 3.02±0.08 | 2.86±0.15 | 0.165 |
| **D28-D35** |  |  |  |  |
| ADG, g/d | 9.37±0.12 | 9.79±0.12 | 10.72±0.26**^**^** | 0.001 |
| ADFI, g/d | 31.17±0.45 | 30.43±0.36 | 30.86±0.11 | 0.344 |
| FCR | 3.33±0.09 | 3.11±0.06 | 2.87±0.08**^**^** | 0.008 |

There were 36 1-day-old Mahuang cocks being chosen in the experiments. They were kept within wired cages with lighting and good ventilation at the 50-55% relative humidity. At 1-14-day old, a 24-h light period was provided for chicken house, followed by gradual decline into 20-h everyday. At 1-7-day-old, chicken house temperature was maintained under 32-34 ℃, followed by gradual decline to 26 ℃. In this experimental process, chickens could eat and drink freely. After the chicks finished acclimatization, the chicks were evenly classified as 3 groups based on body weight at 7 days of age, with 3 chicks for every replicate, and 4 replicates for every group, specifically into: blank control group (NC), 0.125%MLP,0.25%MLPgroup, and the experimental days were 35 days. During this period, NC groups were given the basal diet, the 0.125%groups added 0.125% MLP to the basal diet, the 0.25%groups added 0.25% MLP to the basal diet, On days 14, 21, 28 and 35, feed intake and body weight were determined to calculate the meat and feed ratio.

Data are expressed as mean±SD. **p*<0.05, ***p*<0.01, ****p*<0.001 compared with NC group.

**Results**

On days 21-28, adding 0.125% MLP and 0.25% MLP can both improve the ADG of chicks, but adding 0.25% has a better effect; There was no significant change in daily feed intake; Adding 0.125% and 0.25% MLP can both reduce the FCR of chicks in terms of feed to meat ratio, but adding 0.25% has a better effect. On days 28-35, adding 0.25% MLP significantly increased the ADG of chicks and significantly reduced their FCR (*p*<0.05). Therefore, considering the above, adding 0.25% MLP was chosen.
